# IV Brazilian Consensus on Rhinitis – an update on allergic rhinitis^☆^^☆☆^

**DOI:** 10.1016/j.bjorl.2017.10.006

**Published:** 2017-11-02

**Authors:** Eulalia Sakano, Emanuel S.C. Sarinho, Alvaro A. Cruz, Antonio C. Pastorino, Edwin Tamashiro, Fábio Kuschnir, Fábio F.M. Castro, Fabrizio R. Romano, Gustavo F. Wandalsen, Herberto J. Chong-Neto, João F. de Mello, Luciana R. Silva, Maria Cândida Rizzo, Mônica A.M. Miyake, Nelson A. Rosário Filho, Norma de Paula M. Rubini, Olavo Mion, Paulo A. Camargos, Renato Roithmann, Ricardo N. Godinho, Shirley Shizue N. Pignatari, Tania Sih, Wilma T. Anselmo-Lima, Dirceu Solé

**Affiliations:** a Universidade Estadual de Campinas (Unicamp), Faculdade de Ciências Médicas, Departamento de Oftalmologia e Otorrinolaringologia, Campinas, SP, Brazil; b Associação Brasileira de Otorrinolaringologia e Cirurgia Crânio-Facial, São Paulo, SP, Brazil; c Universidade Federal de Pernambuco (UFPE), Faculdade de Medicina, Departamento de Pediatria, Recife, PE, Brazil; d Sociedade Brasileira de Pediatria, Rio de Janeiro, RJ, Brazil; e Universidade Federal da Bahia (UFBA), Faculdade de Medicina, Departamento de Pediatria – Instituto da Criança, Salvador, BA, Brazil; f Associação Brasileira de Alergia e Imunologia, São Paulo, SP, Brazil; g Universidade de São Paulo (USP), Faculdade de Medicina, Department of Pediatrics – Instituto da Criança, São Paulo, SP, Brazil; h Universidade de São Paulo (USP), Faculdade de Medicina de Ribeirão Preto (FMRP), Departamento de Oftalmologia, Otorrinolaringologia e Cirurgia de Cabeça e Pescoço, Ribeirão Preto, SP, Brazil; i Universidade do Estado do Rio de Janeiro (UERJ), Faculdade de Medicina, Departamento de Pediatria, Rio de Janeiro, RJ, Brazil; j Universidade de São Paulo (USP), Faculdade de Medicina, Departamento de Medicina – Divisão de Imunologia Clínica e Alergia, São Paulo, SP, Brazil; k Universidade de São Paulo (USP), Faculdade de Medicina, Divisão de Otorrinolaringologia, São Paulo, SP, Brazil; l Universidade Federal do Paraná (UFPR), Departamento de Pediatria, Curitiba, PR, Brazil; m Universidade Federal da Bahia (UFBA), Faculdade de Medicina, Departamento de Pediatria, Salvador, BA, Brazil; n Universidade Cidade de São Paulo (UNICID), Faculdade de Medicina, São Paulo, SP, Brazil; o Hospital Sirio-Libanês, Núcleo de Otorrinolaringologia, São Paulo, SP, Brazil; p Universidade Federal do Estado do Rio de Janeiro (UNIRIO), Departamento de Medicina, Divisão de Alergia e Imunologia, Rio de Janeiro, RJ, Brazil; q Universidade Federal de Minas Gerais (UFMG), Departamento de Pediatria, Divisão de Pneumologia, Belo Horizonte, MG, Brazil; r Universidade Luterana do Brasil (ULBRA), Departamento de Otorrinolaringologia, Canoas, RS, Brazil; s Pontifícia Universidade Católica de Minas Gerais (PUC-MG), Instituto de Ciências Biológicas e da Saúde, Belo Horizonte, MG, Brazil; t Universidade Federal de São Paulo (UNIFESP), Escola Paulista de Medicina, Departamento de Otorrinolaringologia, São Paulo, SP, Brazil; u Universidade de São Paulo (USP), Faculdade de Medicina, Departamento de Medicina Legal, Ética Médica e Medicina Social e do Trabalho, São Paulo, SP, Brazil; v Universidade Federal de São Paulo (UNIFESP), Escola Paulista de Medicina, Departamento de Pediatria – Divisão de Alergia, Imunologia Clínica e Reumatologia, São Paulo, SP, Brazil

**Keywords:** Consensus, Rhinitis, Allergic rhinitis, Consenso, Rinite, Rinite alérgica

## Abstract

**Introduction:**

The guidelines on allergic rhinitis aim to update knowledge about the disease and care for affected patients. The initiative called “Allergic Rhinitis and its Impact on Asthma”, initially published in 2001 and updated in 2008 and 2010, has been very successful in disseminating information and evidence, as well as providing a classification of severity and proposing a systemized treatment protocol. In order to include the participation of other medical professionals in the treatment of allergic rhinitis, it is important to develop algorithms that accurately indicate what should and can be done regionally.

**Objective:**

To update the III Brazilian Consensus on Rhinitis – 2012, with the creation of an algorithm for allergic rhinitis management.

**Methods:**

We invited 24 experts nominated by the Brazilian Association of Allergy and Immunology, Brazilian Association of Otorhinolaryngology and Head and Neck Surgery and Brazilian Society of Pediatrics to update the 2012 document.

**Results:**

The update of the last Brazilian Consensus on Rhinitis incorporated and adapted the relevant information published in all “Allergic Rhinitis and its Impact on Asthma” Initiative documents to the Brazilian scenario, bringing new concepts such as local allergic rhinitis, new drugs and treatment evaluation methods.

**Conclusion:**

A flowchart for allergic rhinitis treatment has been proposed.

## Resumo

**Introdução:**

As diretrizes sobre rinite alérgica visam atualizar os conhecimentos sobre a doença e os cuidados para com esses pacientes. A iniciativa designada “Rinite Alérgica e seu Impacto na Asma”, cujo relatório inicial foi publicado em 2001 e atualizada em 2008 e 2010, tem sido muito bem sucedida na disseminação de informações e evidências, bem como na formulação da classificação de gravidade e proposta de sistematização do tratamento. Entretanto, visando a participação de outros profissionais médicos no atendimento da rinite alérgica, é importante o desenvolvimento de algoritmos que indiquem com precisão o que deve e pode ser feito regionalmente.

**Objetivo:**

Atualizar o III Consenso Brasileiro sobre Rinites-2012, com elaboração de algoritmo para conduta da rinite alérgica.

**Método:**

Foram convidados 24 especialistas indicados pelas Associação Brasileira de Alergia e Imunologia, Associação Brasileira de Otorrinolaringologia e Cirurgia Cérvico-Facial e Sociedade Brasileira de Pediatria para atualização do documento de 2012.

**Resultados:**

A atualização do último Consenso Brasileiro sobre Rinites, incorporou e adaptou para a realidade brasileira as informações relevantes publicadas em todos os documentos da Iniciativa “Rinite Alérgica e seu Impacto na Asma”, trazendo novos conceitos como a rinite alérgica local, novos medicamentos e métodos de avaliação de tratamento.

**Conclusão:**

Proposto um fluxograma de tratamento para a rinite alérgica.

## Importance of guidelines for allergic rhinitis management

Guidelines for the treatment of allergic rhinitis (AR) developed over the past 20 years have better elucidated the care for patients with the disease. An expert workshop conducted by the World Health Organization (WHO) in December 1999 led to an initiative called “Allergic Rhinitis and its Impact on Asthma” (ARIA), of which initial evidence-based report had nearly 3000 references and was published in 2001.^1^ An update of the ARIA report was published in 2008, based on new evidence.^2^ This new report resulted from an ongoing review of the literature on previously uncovered aspects, such as: Complementary and Alternative Medicine, sports, update on the associations between rhinitis and asthma, and prevention and treatment.

However, it is necessary to have clear information on the guidelines to facilitate their understanding and acceptance. The ARIA Guide was the first guideline for chronic respiratory disease to use the classification of the Grading of Recommendations, Assessment, Development and Evaluations (GRADE) system, an advanced method that has been adopted by the WHO. A new ARIA review was published in 2010,^3^ ten years after the publication of the first report of the ARIA-WHO workshop. The ARIA Initiative has been very successful in disseminating information and evidence, as well as providing a classification of severity and proposing a systemized treatment protocol. However, this is insufficient to guide new practices, especially when they involve the participation of family physicians, pediatricians, and other health professionals. It is necessary to develop algorithms that accurately indicate what should and can be done for a specific case in your region. A recent publication of experts from the ARIA Initiative proposes a decision-making algorithm in clinical practice for AR control in adolescents and adults.^4^, ^5^

The update of the Brazilian Consensus on Rhinitis – 2017 incorporates and adapts to the Brazilian reality the relevant information published in all ARIA Initiative documents, as was done in previous versions of the Brazilian document,^6^, ^7^ that were always created by representatives of the Brazilian Association of Allergy and Immunology (*Associação Brasileira de Alergia e Imunologia*) and the Brazilian Association of Otorhinolaryngology and Head and Neck Surgery (*Associação Brasileira de Otorrinolaringologia e Cirurgia Cérvico-Facial*) and the Brazilian Society of Pediatrics (*Sociedade Brasileira de Pediatria*).

The main updates made in the base document of 2012^7^ were thus established.

## Definition of rhinitis

Rhinitis is an inflammation and/or dysfunction of the nasal mucosa characterized by some of these nasal symptoms: nasal obstruction, anterior and posterior rhinorrhea, sneezing, nasal pruritus and hyposmia. Symptoms usually occur for two or more consecutive days for more than 1 h on most days.^8^

## Rhinitis classification

Classification reflects the criteria utilized (clinical data, frequency and intensity of symptoms, nasal cytology, etiological factors, and phenotypes [clinical, temporal pattern, severity, duration, control, treatment response, and presence of comorbidities]).^9^

In a recent document, the European Academy of Allergy and Immunology proposed a classification of chronic rhinitis based on the main etiological agent. It consists of 4 subgroups: (1) infectious rhinitis, (2) allergic rhinitis, (3) non-infectious, non-allergic rhinitis, and (4) mixed rhinitis. Infectious rhinitis is acute and self-limiting, and is usually caused by viruses, and less frequently by bacteria. Allergic rhinitis is the most common form and is induced by inhalation of allergens in sensitized individuals. Non-infectious, non-allergic rhinitis represents a heterogeneous group of patients with no signs of infection and no systemic signs of allergic inflammation; examples include drug-induced rhinitis, rhinitis of the elderly, hormonal rhinitis, gestational rhinitis, occupational non-allergic rhinitis, gustatory rhinitis, and idiopathic rhinitis. Mixed rhinitis occurs in patients with chronic rhinitis who have more than one etiological agent, known or unknown.^10^

Another recent concept is that of the endotype that aims to identify the underlying mechanisms involved in the genesis of the disease, thus allowing targeted and more accurate treatment for each patient.^9^, ^10^ These endotypes are complex and secondary to cell processes (eosinophils, neutrophils and inflammatory mediators caused by them), molecular (total and specific serum IgE, inflammatory cytokines and chemokines) and structural damage of the nasal mucosa.^9^, ^10^ To date, four rhinitis endotypes have been identified: (a) with type 2 immune response; (b) with type 1 immune response; (c) neurogenic rhinitis; (d) epithelial dysfunction (9). A better characterized rhinitis can receive an individualized and more specific treatment, with a greater chance of success.

## Allergic rhinitis

The classification of the ARIA guide, based on symptom frequency and intensity, was maintained due to its great acceptance in the Brazilian medical scenario.^1^

### Prevalence

As observed in several parts of the world, the International Study of Asthma and Allergic Diseases in Childhood carried out in several Brazilian locations, has shown an increase in the prevalence of nasal symptoms among children and adolescents in the last year, reaching 37.2% (oscillating between 26.3% and 49.9%) and 16.2% (ranging from 15.4% to 27.9%) for allergic rhinoconjunctivitis.^11^

### Clinical picture

The clinical picture remains important for the diagnosis of allergic rhinitis. In addition to the characteristic symptoms (sneezing, itching, rhinorrhea and nasal obstruction) obtaining a personal and comprehensive allergic history is crucial, as well as identifying any triggering factors.^12^

### Triggering factors

National studies have reinforced the involvement of house dust mites as the main etiological agents of allergic rhinitis, followed by cockroach, pet epithelium and, more rarely, fungal allergens.^13^, ^14^ In the southern region of the country, pollen is an important factor in the sensitization of adults and children.^15^ The role of mucosal irritants is reinforced, with special emphasis on pollutants and irritants (Table 1).^16^

**Table 1:** Triggering factors for respiratory allergies.^16^

*Aeroallergens*	
House dust mites	*Dermatophagoides pteronyssinus*, *Dermatophagoides farinae*, *Blomia tropicalis*
Cockroaches	*Blattella germanica*, *Periplaneta americana*
Fungi	*Aspergillus sp*, *Cladosporium sp*, *Alternaria sp, Penicillium notatum*
Pets	Cats, dogs, rabbits, horses and rodents (hamster, guinea pig, domestic ferret, mice)
Pollens	Grasses – Lolium multiflorum (ryegrass), Phleum pratense
Occupational	Wheat, wood dust, detergents, latex

*Pollutants*	
Intra-domiciliary	Cigarette smoke, particulate matter (PM 10) and nitrogen dioxide (NO 2) derived from cooking gas or wood stove combustion
Extra-domiciliary	Ozone, NOx and sulfur dioxide

*Irritant agents*	Strong odors, perfumes, air conditioning, cleaning products

Foods rarely trigger respiratory symptoms alone. Most often they occur as manifestations associated with more severe conditions, such as anaphylaxis.^17^

### Pathophysiology

Cellular and molecular mechanisms involved in the allergic reaction and responsible for allergic inflammation are shown to facilitate the understanding of the different proposed endotypes of rhinitis.^9^

### Diagnostic resources

According to the purpose of evaluation they are divided into: (a) etiological diagnosis, (b) nasal cavity assessment, (c) imaging assessment and (d) complementary assessment.

a) Etiological diagnosis
  The most important subsidiary exams in the etiological diagnosis of allergic rhinitis, both for specificity and sensitivity, are the skin prick test (SPT) and the evaluation of serum levels of allergen-specific IgE. The diagnosis of allergy and the identification of the most relevant allergens in each case are important for targeted preventive interventions, such as environmental control, pharmacological treatment options and, finally, the possibility of specific immunotherapy with allergens.
  The SPTs with aeroallergens are the most commonly used tests in the diagnosis of respiratory allergy and portray IgE-mediated allergic reactions. They should preferably be performed with standardized allergens, chosen according to the clinical relevance of the patient's history, age, profession, environment, and regional distribution of allergens; they should be performed under direct supervision of an appropriately trained physician to avoid false-positive and false-negative results and potential systemic reactions.^18^ The reaction intensity is generally reduced in the extremes of life and in the presence of extensive eczema or dermographism. The use of oral antihistamines and of topical corticosteroids in the preceding 7 days are contraindications for the test.^18^
  Although high levels of total serum IgE are recognized by some authors as synonymous with allergic disease, they can also be detected in different diseases such as HIV infection, allergic pulmonary aspergillosis, allergic fungal sinusitis, lymphomas, tuberculosis, parasitic diseases with a pulmonary cycle, and others. Thus, its significance in the diagnosis of allergies is limited. Similar to the SPT, total serum IgE levels are low in the first years of life.^19^
  On the other hand, the presence of serum specific IgE to a particular allergen is a strong indicator of allergic sensitization, but should not be considered in the absence of allergic symptoms.^20^, ^21^ It has a sensitivity and specificity similar to those of SPT, but it is more expensive and requires a venepuncture, specialized laboratory techniques and takes longer to obtain the result. However, it is not affected by medications and skin conditions and does not harbor the risk of severe allergic reactions. It can be used to evaluate a greater number of allergens, has better reproducibility and is not affected by the test technique.^20^
  More recently, the acquisition of Component Resolved Diagnostic (CRD) allowed the determination of specific IgE to multiple allergens (recombinant or not) using the microarray technique (*e.g.*, Immuno-solid-phase allergen chip (ISAC) assay) allowing greater diagnostic precision and the possibility to discriminate cross-sensitization co-sensitization by different allergens that have the same protein in their composition.^21^, ^22^
  In Brazil, ImmunoCap-ISAC (ThermoFisher Scientific), a multiple platform that identifies 112 natural or recombinant components from 51 allergenic sources is available. Its high cost still limits its use to very special cases.
  Although nasal provocation tests (NPTs) are more commonly used in the research setting, they have shown to be useful in allergic rhinitis diagnosis, including local allergic rhinitis, and non-allergic rhinitis.^23^, ^24^, ^25^ They are useful in the diagnosis of occupational rhinitis, aiming to identify and quantify the clinical relevance of inhaled allergens or occupational irritants.^23^, ^24^ NPTs should be performed by specialized physicians at appropriate sites and using standardized allergen extracts; the assessment of nasal response may be performed by symptom score, but the use of the objective monitoring method is recommended.^23^, ^24^
b) Nasal cavity assessment
  Other examinations such as nasal cytology, bacterioscopy, and culture of airway secretions, olfaction evaluation, nasal permeability (by acoustic rhinometry, rhinomanometry, or peak nasal flow) are used less frequently.^12^
c) Imaging assessment
  The plain rhinopharynx radiograph is useful for the diagnosis of nasal obstruction by pharyngeal tonsil hypertrophy (adenoid) or other rhinopharyngeal tumor processes. The paranasal sinuses radiographs (Waters’ and Caldwell's Views) have no role in the diagnosis of allergic rhinitis.^26^
  Computed tomography and magnetic resonance imaging of the paranasal sinuses may be necessary in the evaluation of chronic inflammatory and infectious sinus conditions, complications of acute infectious conditions, and evaluation of benign and malignant tumor processes.^26^
d) Complementary assessment
  It is known that allergic rhinitis has a high impact on the lives of patients and their families. Recently, self-administered questionnaires have been created to allow a broader evaluation of patients and allow a more personalized and comprehensive therapeutic approach. The most important are those that evaluate sleep disorders and those that assess quality of life.
  Sleep-related disorders (SRDs) are associated with environmental, social, cultural, biological, family and emotional factors and are generally underreported and often not considered and investigated.^27^, ^28^ Polysomnography is the gold standard for the diagnosis of SRDs and is an objective evaluation; however, it is expensive and difficult to perform, which limits its use in population studies. Sleep diaries are inexpensive, but require time and compliance to complete, and are difficult to interpret.^28^
  Recently, written questionnaires have been developed to evaluate SRDs in different age groups. They are self-administered and easy to apply, as well as low-cost and useful for use in large studies.^29^, ^30^ One example is the Children's Sleep Habits Questionnaire (CSHQ).^31^ A recent study documented a significantly higher CSHQ score in children with asthma and/or rhinitis compared to healthy children, especially those with poorly controlled disease.^32^
  Other tools that have been increasingly used in the follow-up of patients with allergic rhinitis are the Health-Related Quality of Life (HRQL) questionnaires that allow a broader assessment of how the disease interferes with the patient's daily life. They are short, self-applied, easy to understand and inexpensive.^33^ Some examples of these questionnaires are the Rhinoconjunctivitis Quality of Life Questionnaire (RQLQ),^34^ the Mini Rhinoconjunctivitis Quality of Life Questionnaire (MiniRQLQ)^35^ and the Pediatric Rhinoconjunctivitis Quality of Life Questionnaire (PRQLQ).^36^

### Comorbidities

The main comorbidities associated with allergic rhinitis are asthma, allergic conjunctivitis, acute and chronic rhinosinusitis, otitis media with effusion and changes in the craniofacial development of oral breathers in children, as well as obstructive sleep apnea and hypopnea, both in children and in adults.^12^

### Treatment

The treatment of the patient with allergic rhinitis includes non-pharmacological and pharmacological measures. Non-pharmacological measures aim to reduce the patient's exposure to irritants and/or agents to which they are sensitized. Although there is debate about the effects of these environmental control measures on the control of respiratory allergies, they have been defended by several researchers.^37^ Table 2 lists the main measures to be taken for effective environmental control.^16^

**Table 2:** Environmental control measures.^16^

– The bedroom should be preferably well ventilated and sunny. Avoid pillows and mattresses made of kapok fibers or feathers and prefer those made of foam, artificial fibers or latex, wherever possible covered by plastic material (vinyl) or mite-impermeable covers. The bed frame should be cleaned twice a month. Bedding and blankets should be changed and washed regularly with detergent and at high temperatures (>55 °C) and dried in the sun or with hot air. If possible, the mattress surface should be vacuumed using a powerful domestic vacuum cleaner model. – Avoid rugs, carpets, curtains and cushions. Give preference to washable floors (ceramic, vinyl and wood) and venetian blinds or shutters instead of curtains, made of material that can be cleaned with a damp cloth. If the carpets or rugs are very heavy and difficult to remove, they should be vacuumed twice a week, if possible, after being allowed to ventilate. – Beds and cribs should not be placed next to the wall. If that is not possible, place it next to the wall with no marks of humidity or next to the sunniest one. – Avoid stuffed animals, book shelves, magazines, cardboard boxes or any other place where dust mite colonies can be formed in the bedroom. Replace them with fabric toys, so they can be washed frequently. – Identify and eliminate mold and moisture, especially in the bedroom, reducing humidity to less than 50%. Periodically check the humid areas of your home, such as bathroom (plastic shower curtains, under the sinks, *etc.*). A diluted bleach solution can be applied to the moldy places until its final resolution, even though they are respiratory irritants. It is essential to investigate other sources of exposure to fungi outside the home (day care, school and workplaces). – Avoid using ordinary brooms, dusters, and vacuum cleaners. Use a damp cloth daily to clean the house or use vacuum cleaners with special filters twice a week. Remove the allergic patient from the environment while cleaning. – Environments that have been closed for prolonged periods (beach or country house) should be ventilated and cleaned at least 24 h prior to the arrival of individuals with respiratory allergy. – Avoid the presence of pet animals and birds, especially in the patient's bedroom and bed (safe environment). Keep the bedroom door always closed. If it is impossible, restrict the animal to a single area of the house and use HEPA filters in the patient's room. Preferably, pets for allergic children should be fish and turtles. – Avoid exposure to mouse and rat allergens, with professional intervention integrated with house cleaning measures; including the placement of traps, sealing of holes and cracks that may serve as entry points and the use of rodenticide in cases of large infestations. – Inspection is an important step for cockroach extermination. Avoid insecticides and cleaning products with strong odors; prefer using the bait method. Exterminating cockroaches and rodents may be necessary. – Remove the garbage and keep food in closed packages, as these attract rodents. Do not store garbage indoors. – Give preference to soap paste and powder versions for bathroom and kitchen cleaning. Avoid talcum powder, perfumes, deodorants, especially in spray. – Do not smoke or allow smoking inside your home or car. Prenatal, perinatal and postnatal smoking is associated with future respiratory problems in the offspring. – Avoid extremely hot baths and sudden temperature fluctuations. The ideal temperature of the water is body temperature. – Prefer the outdoor life. Sports can and should be practiced, avoiding days with high exposure to pollen or pollutants in certain geographic areas. – Patients allergic to pollen are advised to keep the house and car windows closed during the day, opening them at night (lower pollen count). House and car ventilation systems should be equipped with special pollen filters. Protective masks and safety glasses are useful. Pollen can be transported indoors on clothing and pets. Avoid leaving clothes to dry in the open air; if possible, use an electric dryer. – Avoid outdoor activities during periods of high pollen counts, between 5 and 10 o’clock in the morning and on hot, windy, and dry days. – Keep air conditioner filters clean. If possible, clean them monthly. Avoid exposure to very low ambient temperature and sudden temperature fluctuations. Remember that the conditioned air is dry and can be irritating.

For pharmacological measures, we highlight the different classes of drugs commonly used in the therapeutic approach of patients with allergic rhinitis: H1 antihistamines alone (anti-H1, systemic or nasal topical), decongestants (systemic, nasal topical), corticosteroids (systemic, nasal topical), disodium cromoglycate, and leukotriene receptor antagonists. In addition to these, saline solution, allergen-specific immunotherapy and, more recently, immunobiological agents have constituted the therapeutic arsenal of patients with allergic rhinitis.^12^

Anti-H1 agents are considered first-line drugs for the treatment of AR, especially the second generation or non-classic drugs.^2^, ^3^, ^7^ As they act on the histamine H1 receptor, they effectively relieve the symptoms of the immediate phase of AR, such as nasal pruritus, sneezing, rhinorrhea and associated ocular symptoms, and improve the nasal obstruction characteristic of the late phase of the disease.^38^ Because they are less lipophilic and have low passage through the blood-brain barrier, they bind poorly to brain H1 receptors and, therefore, cause fewer adverse effects on the central nervous system, such as sedation.^39^

Second-generation anti-H1s have a rapid onset of action; the duration of use of these medications varies from 1 to 4 weeks, but they may be used for prolonged periods of time in moderate to severe and persistent cases. Due to their excellent safety profile and therapeutic advantages in the treatment of allergic rhinitis, second-generation anti-H1 should always be prescribed, rather than older compounds for all age groups.^40^, ^41^, ^42^

In addition to oral formulations, antihistamines are currently available for nasal and topical ophthalmological use. Nasal topical anti-H1 have similar efficacy to the oral compounds, and have as a therapeutic advantage a faster onset of action and greater effectiveness in controlling nasal obstruction.^43^, ^44^, ^45^

Nasal decongestants are adrenergic or adrenomimetic stimulants with a principal action of vasoconstriction, resulting in rapid relief of nasal obstruction in allergic rhinitis.^46^ They are divided into two groups: oral and nasal topical use. Pseudoephedrine is the most commonly used oral decongestant, followed by phenylephrine. In Brazil, these are only available in combination with an anti-H1 agent. Pseudoephedrine should be used with caution due to its psychotropic action and potential cardiovascular side effects. It is not recommended for patients younger than 4 years of age because of the increased risk of toxicity, and prolonged release formulations at doses of 120 mg are not recommended for children under 12 years of age.^46^, ^47^ Topical nasal decongestants should be used at maximum for up to 5–7 days, as prolonged use increases the risk of drug-resistant rhinitis, very often a difficult problem to resolve. Furthermore, they can cause important cardiovascular effects, as well as affect the central nervous system (imidazole derivatives); they are contraindicated in children below six years of age. They should also be avoided in the elderly, due to the higher incidence of hypertension and urinary retention resulting in this age group.^46^

The combination of oxymetazoline and mometasone furoate for nasal topical use achieved a rapid onset of action, better efficacy on nasal obstruction, and a reduction in polyp size in patients with seasonal allergic rhinitis and nasal polyposis, compared to the two drugs administered separately.^48^, ^49^

Corticosteroids, which are potent anti-inflammatory agents, have been widely used in the treatment of several diseases, including allergies. In patients with allergic rhinitis, systemic corticosteroids are reserved for patients with severe exacerbations or severe forms of allergic rhinitis, and always for a short period of time (5–7 days) to prevent adverse effects resulting from prolonged use.^2^, ^12^ However, parenteral administration of prolonged action corticosteroids (known as depot),) is contraindicated in the management of rhinitis, especially in children and the elderly, due to adverse systemic effects.^12^ A recent study evaluated the action of the combination of Desloratadine (0.5 mg/mL) and prednisolone (4 mg/mL) for 7 days in children (2–12 years) with a severe acute crisis of allergic rhinitis and documented significant symptom control in the first 24 h accompanied by a lower incidence of adverse events, especially drowsiness.^50^

Nasal corticosteroids (NCs) for topical use have a broader safety profile, that allows them to be used for longer periods of time and are the anti-inflammatory treatment of choice recommended by most specialists treating allergic rhinitis (from several medical societies^1^, ^2^, ^3^, ^8^, ^12^, ^51^). Nasal corticosteroids for topical use have also been shown to be effective in controlling occupational, gestational, and idiopathic rhinitis.^51^

NCs improve nasal congestion, olfactory alterations, rhinorrhea, sneezing, nasal pruritus and associated ocular symptoms secondary to a possible action on the naso-ocular reflex (allergic rhinoconjunctivitis). Their use results in the improvement of quality of life, quality of sleep and daytime concentration. Treatment with NCs also reduces the risk of complications such as rhinosinusitis, secretory otitis and asthma.^1^, ^2^, ^12^

In Brazil the available NC formulations are beclomethasone dipropionate (BDP), budesonide (BUD), fluticasone propionate (FP), mometasone furoate (MF), fluticasone furoate (FF) and ciclesonide (CIC). The NCs approved for use in individuals older than two years of age are MF and FF; for those older than 4 years of age, they are BUD and FP, while BDP and CIC are reserved for those older than 6 years.^12^ NC onset of action occurs 7–12 h after administration, but patients should be advised that the final therapeutic benefit may take up to 14 days.^12^, ^51^ Although all of the agents have an anti-inflammatory action, they differ in their pharmacodynamic and pharmacokinetic characteristics, which gives them different safety profiles.^51^

Studies show that the therapeutic effect of NCs depends not only on the effectiveness of the active substance, but also on the deposition of the product in the nasal cavity (spray or aerosol),^51^, ^52^ their affinity for the glucocorticoid receptor, and the concentration–time association at the site of action and lipophilicity, which are important factors for both the therapeutic effect and the potential to reach the systemic circulation (systemic availability) for its elimination.^51^, ^52^ It is believed that the ideal NCs should have high lipophilicity, low systemic availability and high systemic clearance.^52^, ^53^

The adverse effects of NC are, for the most part, dependent on serum availability, which is decreased by the ability of the drug to bind to plasma proteins. The new generation of NCs exhibits high affinity for plasma protein binding which affects its systemic bioavailability: FF, FM and CIC bind in 99%; FP in 90%, BUD in 88% and BDP in 87%.^53^, ^54^

The main adverse effects related to the use of NCs are local (irritation, bleeding, septal perforation) and can be observed with any of the drugs used and are dependent on the dosage and the technique of administration. Systemic adverse effects (interference with the hypothalamic-pituitary-adrenal axis, ocular effects, effects on growth, bone resorption and skin effects), can vary according to the patient's age, dosage and drug pharmacokinetics.^53^, ^54^

Although indicated as safe, even on a small scale, all NCs are absorbed systemically in part and may exhibit adverse effects. The effects of NCs in children and in pregnant women are similar to those observed in adult patients.^54^ However, the use of NCs in pregnant women requires greater consideration, since there is always concern about embryogenesis. BUD is the only NC that falls into category B for use in pregnancy, mandating a prescription at the smallest possible dose and duration.^55^

The combination of anti-H1 (azelastine hydrochloride) and NC (fluticasone propionate) for topical use in a single dispenser was initially recommended only for patients over 12 years of age who had persistent moderate or severe symptoms not controlled by an anti-H1 and/or NC.^55^, ^56^, ^57^, ^58^ It has now been shown to be effective and safe in children aged four years and older.^59^, ^60^

Studies in patients with allergic rhinitis have compared treatments with the combination of the two drugs to the drugs administered separately and found that the combination was more effective in the control of these patients,^55^, ^56^, ^57^, ^58^, ^59^, ^60^ and there was no loss of effectiveness even when they were administered for prolonged periods of time.^61^ The incidence of adverse events has been similar to that observed with patients treated with placebo. To date, there are insufficient data on their safety in pregnant or breastfeeding women.^55^, ^61^

Disodium cromoglycate has a stabilizing action on the membrane of mast cells and, consequently, prevents the release of their chemical mediators during the allergic reaction. They are superior to placebo, but much less effective compared to anti-H1 and NCs in the control of rhinorrhea, sneezing and nasal pruritus, in addition to having little effect on nasal obstruction.^2^, ^62^ The drug has an excellent safety profile, making it an acceptable therapeutic alternative in infants, an age group for which NCs are not approved.^2^, ^62^ Due to its short half-life, it is administered 4 to 6 times a day, which makes treatment adherence difficult.

Montelukast sodium (MS) is the only compound representative of leukotriene receptor antagonists available in Brazil. It is superior to placebo in symptom control and quality of life improvement of patients with allergic rhinitis.^63^, ^64^, ^65^ Although MS is not the first choice for the treatment of patients with allergic rhinitis, it has been suggested as a therapeutic alternative for patients with concomitant asthma and allergic rhinitis^66^ and in those with difficulty adhering to treatment regimens using topical nasal medication. Additionally, they can be considered in cases of chronic rhinosinusitis with nasal polyposis, and in aspirin-exacerbated respiratory disease (AERD).

Recently an association between an anti-H1 (levocetirizine 5 mg + montelucaste de sódio 10 mg) has been made available for individuals older than 18 years.^67^ Studies in adults have shown that the combined therapy is superior to both medications when given alone.^68^, ^69^, ^70^

Allergen-specific immunotherapy (SIT) remains the only treatment that can modify the allergic disease.^71^ Additionally, it provides long-lasting benefits after its discontinuation,^72^ prevents the progression of disease, including the development of asthma,^73^, ^74^ as well as the development of new sensitizations.^75^, ^76^, ^77^

SIT is recommended for the treatment of adults and children (>5 years) with intermittent moderate/severe allergic rhinitis and in all its persistent forms,^77^ always by a specialist in allergology. The indication of SIT must be based on the evidence of specific allergen sensitization by *in vivo* or *in vitro* methods, the relevance of the allergen (s) for symptom onset, the impossibility of avoiding exposure to the allergen (s) and the availability of standardized and confirmed effective allergen extract.^12^, ^78^ It is a long-term therapeutic procedure.

The following are absolute contraindications to SIT: uncontrolled asthma, active autoimmune disease, malignant neoplasm, children under two years old, and patients with human immunodeficiency virus (HIV) infection. The following are relative contraindications: partially controlled asthma, autoimmune disease in remission, use of beta-blockers, cardiovascular diseases, children between 2 and 5 years of age, HIV infection (classification A and B, CD4 > 200 cells/mm^3^), chronic infections, immunodeficiencies and use of immunosuppressants.^79^

Nasal lavage with saline solution has been used as an adjuvant in the treatment of acute and chronic nasal conditions. As it is an inexpensive, practical and well-tolerated method, it became very widespread. The use of saline solution facilitates the removal of secretions, thus promoting symptomatic relief to patients.^51^ In the specific case of inflammatory and allergic rhinitis, nasal lavage also promotes the removal of inflammatory mediators present in the nasal mucus, therefore improving the clinical picture.^51^ Isotonic saline should be used 1–2× daily as adjunct treatment for allergic rhinitis.^80^ Clinical observation recommends prior nasal lavage before the administration of other nasal topical medications.

Biological agents (human or humanized monoclonal antibodies) developed for the treatment of severe asthma have been used with good results in other diseases, such as chronic urticaria, chronic rhinosinusitis, nasal polyposis and allergic rhinitis. They have been synthesized by living organisms and directed against a specific target, for instance, a cytokine or its receptor.^81^ The identification of different molecular pathways that have clinical significance helped establish the treatment targets and led to the identification of the described endotypes in asthmatics and that could well be transferred to allergic rhinitis.^82^

In allergic diseases the targets against which biological agents have been developed are: IgE, Th2-response cytokines, such as IL-4, IL-5, IL-9, IL-13, IL-31, and TSLP, CCR4 chemokine receptor, and surface adhesion molecules CD2, CD11a, CD20, CD25, CD52, and ligand OX40. However, there is little evidence of the use of biologicals in allergic rhinitis.

A meta-analysis evaluated the efficacy and safety of omalizumab in patients with uncontrolled allergic rhinitis and verified that it achieved significant symptom relief, reduced use of rescue medication, and quality of life improvement in these patients.^83^ Omalizumab is generally well tolerated and its re-administration is not followed by the formation of antibodies against the medication and, therefore, it is not immunogenic.^84^ Moreover, the addition of anti-IgE agents to specific allergen immunotherapy reduced the rate of systemic reactions to SIT.^85^, ^86^, ^87^

Biologicals including anti-IL-5 (mepolizumab, reslizumab), anti-IL4/13 (dupilumab) were studied in patients with different conditions, but efficacy was not verified in all patients. The target of these biologicals are specific molecules that participate in the pathogenetic mechanisms of asthma, rhinitis, atopic dermatitis and chronic rhinosinusitis.^88^ What is expected is the disease control through the reduction of immunological inflammation and production of IgE antibodies. Studies designed to evaluate the action of these therapeutic resources having allergic rhinitis as the primary outcome are required.^89^

### Clinical control assessment

Similar to what was observed in several chronic diseases, such as asthma and chronic urticaria, the concept of clinical control in rhinitis has been appreciated in recent years. This concept can be defined as the level at which the disease management objectives are achieved by the implemented treatment.^90^

In contrast to the disease severity level, a criterion classically used to define the treatment of rhinitis,^2^ rhinitis control seems to be a more adequate criterion to guide its treatment.^90^ However, the assessment of disease control should be personalized and there is no substitute for adequate follow-up of allergic rhinitis in the context of the physician–patient relationship. Several tools have been developed with the aim of assisting the evaluation of rhinitis control by physicians and/or in the screening of patients not controlled in the primary care.^90^, ^91^ These include the visual analog scale (VAS) and assessment questionnaires/scores. The VAS has recently been proposed by some medical organizations and societies as a tool for routine patient self-evaluation and as an auxiliary method for the pharmacological treatment management.^4^, ^92^, ^93^

The most recently proposed control assessment questionnaires/scores differ in the focus given to the concept of control, either by more intensely addressing the disease symptoms, or by assessing the impact of the consequences of rhinitis on activities of daily living. Additionally, specific questionnaires for the evaluation of rhinitis and questionnaires that address both rhinitis and asthma have been developed. These include: Rhinitis Control Assessment Test (RCAT)^94^, ^95^; Rhinitis Control Scoring System (RCSS)^96^ and the Allergic Rhinitis Control Test.^97^ The Control of Allergic Rhinitis and Asthma Test (CARAT) was designed to jointly evaluate the control of adolescent and adult patients with asthma and rhinitis^98^ and the Control of Allergic Rhinitis and Asthma Test – Kids (CARATkids) for children aged 6–12 years.^99^

### Surgical treatment

Surgical treatment of allergic rhinitis aims to correct the associated chronic nasosinusal anatomical changes. This is especially true for patients with nasal obstruction refractory to clinical treatment and those who exhibit inferior turbinate hypertrophy.^41^ The benefits reported by observational studies indicate potential improvement in breathing and consequent improvement in quality of life, as well as better distribution of topical medications in the nasal cavity.^40^

To date, no technique has been established as the gold standard. The selection of the technique to be employed is individualized and depends on factors such as: greater or lesser boney or mucosal components of the inferior turbinate, surgeon's experience, available equipment, and cost, among others.^100^

## Final considerations

**Figure 1:**
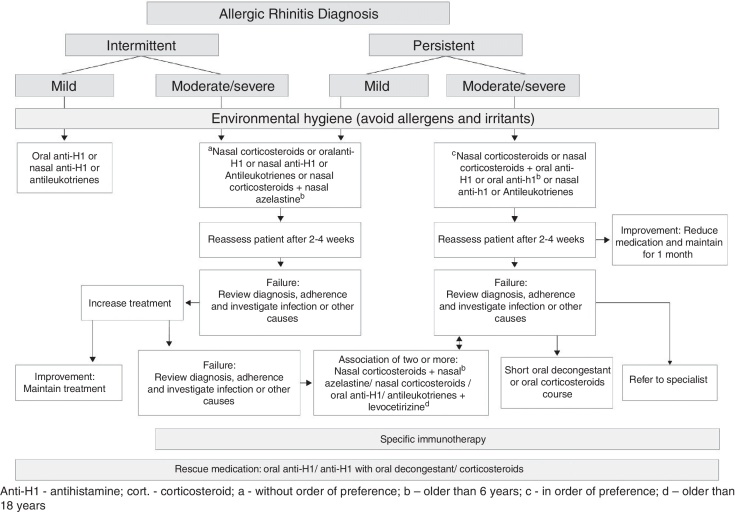
Treatment flowchart for allergic rhinitis.

Based on the recommendations for the treatment of allergic rhinitis published by the ARIA initiative^2^, ^4^, ^5^ and by the European Academy of Allergy and Immunology and the American Academy of Asthma, Allergy and Immunology^9^ and the American Academy of Otorhinolaryngology,^40^ we proposed the flowchart for the treatment of allergic rhinitis (Fig. 1).

## Conflicts of interest

The authors declare no conflicts of interest.
